# Students’ perspective on new teaching concepts for medical studies: case- and competency-based learning in radiology

**DOI:** 10.1186/s13244-025-01909-7

**Published:** 2025-02-06

**Authors:** Max Masthoff, Friedrich Pawelka, Gisela Zak, Bas de Leng, Dogus Darici, Philipp Schindler, Walter Heindel, Anne Helfen

**Affiliations:** 1 Clinic for Radiology, University and University Hospital of Münster, Münster, Germany; 2 Educational Institute (IfAS), Faculty of Medicine, University of Münster, Münster, Germany; 3 Institute of Anatomy and Molecular Neurobiology, University of Münster, Münster, Germany

**Keywords:** Case-based learning, Competency-based learning, Radiology teaching, Medical studies

## Abstract

**Objective:**

This study aimed to evaluate medical students’ perception of a new radiology teaching format for abdominal diagnostics. The format transitioned traditional lectures and seminars to a case- and competency-based course that incorporates technology-enhanced individual case-work, small group discussions, and concise lectures.

**Materials and methods:**

235 students (23.5 ± 2.6 years, 72.3% female, 93.3% response rate, November 2023–June 2024) completed a questionnaire before (12 items) and after (20 items) the course, assessing perceived importance of course content, competency gains in abdominal imaging, enjoyment of learning, interest in a radiology career, and pedagogical perception of the teaching concept. Responses were recorded on a 1–10 scale (no agreement to strong agreement) or dichotomously (yes/no). The new course format was compared with a cohort of students who had previously (May 2022–June 2023) attended traditional lectures (*n* = 169) and/or seminars (*n* = 234).

**Results:**

Students strongly agreed before the course that radiology content in abdominal diagnostics is important, and they found the content highly relevant and applicable to their work as doctors following the course. Significant improvement was observed in perceived competency in modality selection and description and interpretation of common pathologies, with the strongest effect for CT and MRI data. The new format was rated more motivating and significantly better in pedagogical and content quality than traditional lectures and seminars, although it did not influence students’ interest in pursuing a radiology career.

**Conclusion:**

From the students’ perspective, case- and competency-based teaching enhances skill acquisition, learning success, and enjoyment in radiology.

**Clinical relevance statement:**

From a student perspective, case- and competency-based teaching in radiology may enhance imaging competency, contributing to the development of more skilled healthcare providers.

**Key Points:**

- Case- and competency-based teaching concepts may improve students’ learning.
- Students reported improved perceived competency in decision-making and image interpretation with the new teaching method.
- Case- and competency-based teaching was perceived as more engaging, motivating, and pedagogically superior to traditional lectures.

**Graphical Abstract:**

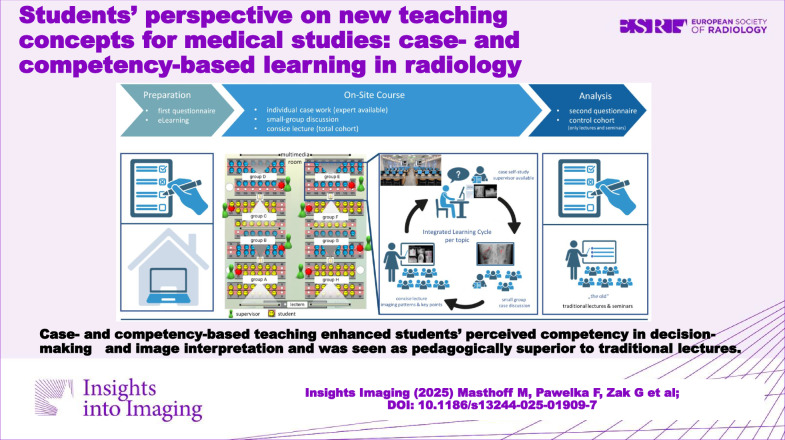

## Introduction

Radiology is a cornerstone of modern medicine, serving as an essential diagnostic and therapeutic discipline in managing a wide range of clinical conditions. Consequently, for physicians across nearly all specialties, a deep understanding of radiological principles—encompassing applied imaging methods, their advantages and limitations, as well as the interpretation of common radiological patterns within the context of disease—is indispensable. Such expertise facilitates the effective exchange of clinical and radiological data across disciplines, ultimately aiming to enhance patient care and treatment outcomes.

Given the importance of radiology in clinical practice, there is a clear need for a resilient education of medical students—our future physicians. This need is increasingly met by a demand for high-quality radiology teaching that emphasizes interactive, case- and problem-based learning rather than traditional, passive lecturing [1–3]. It has been shown that problem-based learning not only fosters long-term knowledge retention and the practical application of skills but also enhances soft skills such as communication and intellectual curiosity when compared to lecture-based learning [4–6].

In response to these educational demands, radiological curricula are being restructured by national authorities and societies to adopt a competency-based framework, prioritizing practical, case-based learning [7, 8]. In this context, the latest version of the Curriculum for Undergraduate Radiological Education, developed by the European Society of Radiology (ESR), emphasizes acquiring knowledge of key imaging techniques and common imaging patterns [9]. Here, it focuses on developing the skills and competencies needed to analyze and interpret imaging data effectively and to communicate results clearly to both patients and clinical colleagues. Despite these advancements, new high-quality, augmented radiology teaching methods remain underutilized, with traditional lecture-based formats still constituting the majority of educational content [5, 10]. Nonetheless, innovative approaches such as the integration of radiology education within gross anatomy and physiology teaching [11], or the implementation of practice-based “smart” classes have been implemented [12–14].

However, there is a paucity of data on how medical students perceive these novel case- and competency-based teaching formats. To address this gap, our study aimed to conduct a comprehensive analysis of students’ perceptions of a modern, case- and competency-based abdominal radiology course, focusing on their views regarding perceived importance and fun, gains in competence, and the overall engagement with the pedagogical concept and radiology.

## Methods

### The “old”—traditional lectures and seminars

Medical students in Germany enter medical school directly after high school [12]. The curriculum includes a 2-year preclinical stage and a 4-year clinical stage, with radiology integrated throughout the latter. Abdominal radiology, taught in the fourth year, traditionally relied on lectures and seminars. Lectures (45–90 min) used PowerPoint (Microsoft Corporation, Redmond, USA) presentations with radiological images and theoretical content, delivered on-site or online. Seminars (90 min) for groups of 20 focused on image-based discussions but had limited active participation. The seventh-semester curriculum included five lectures and one seminar. Further details are provided in the Supplementary methods.

### The “new”—case- and competency-based radiology teaching

**Fig. 1:**
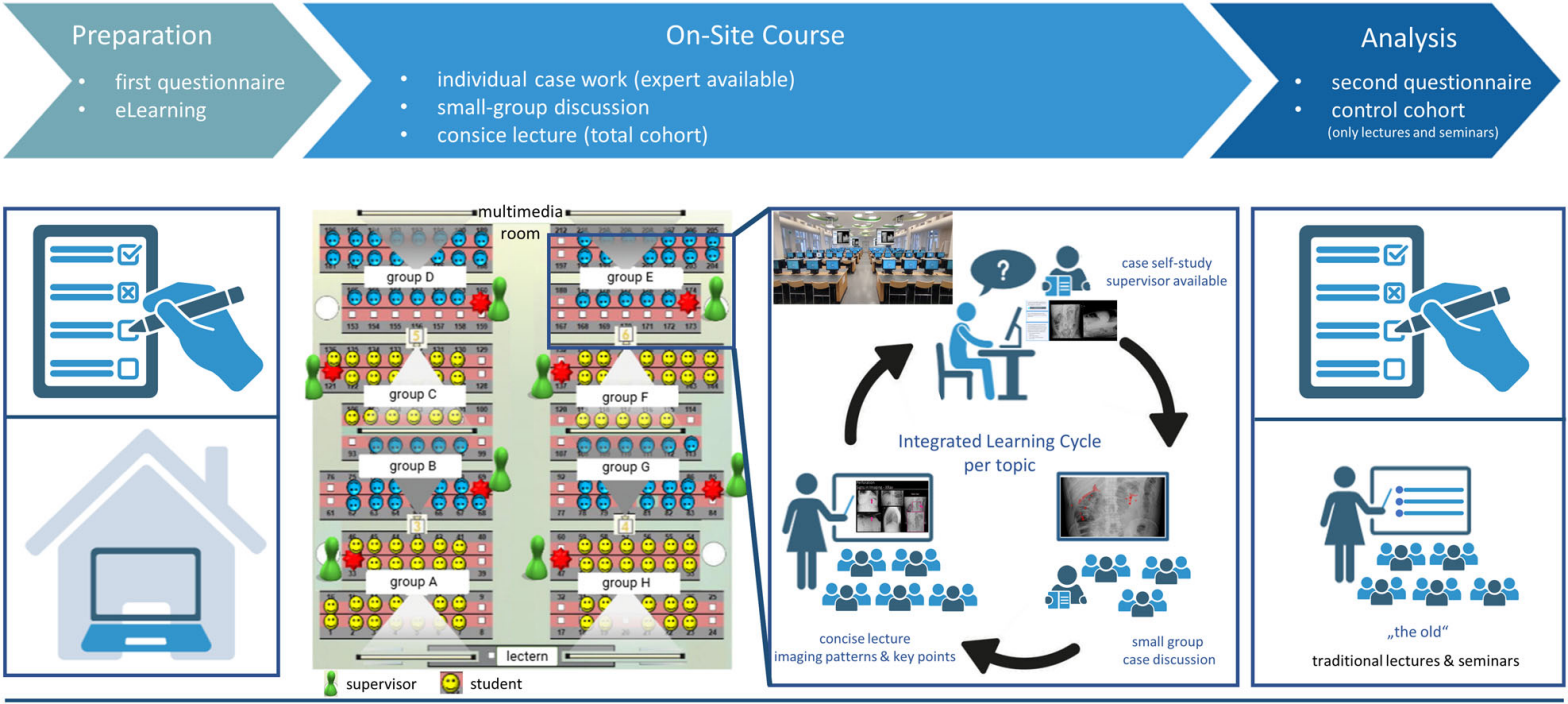
Scheme of the new case- and competency-based teaching course and the study design. Figure partially created using biorender.com

The new concept for teaching radiology is built on a case- and competency-based framework settled within a day course on radiology [15]. A scheme of the study design and the new radiology course, structured around an integrated learning cycle that includes self-study, small group discussions, and concise lectures, is presented in Fig. 1.

Within a week in advance to the on-site course, students have to attend a preparatory eLearning module. The eLearning module is mandatory for all students and contained basic information on imaging modalities and contrast phases used for examination of the abdomen and a repetition of gross anatomy in imaging (x-ray, computer tomography (CT) and magnetic resonance imaging (MRI)), which was already content of a mandatory curricular anatomy-radiology cross-link course in the third semester of the “preclinical” stage [11].

The on-site day course (8 h, including breaks of 1.5 h in total) is attended by the entire cohort of students and is mandatory. This course starts with a short introductory lecture on technical aspects as well as information on patient preparation and radiation protection for radiological examinations of the abdomen. In the following the day is divided into eight modules, each comprised of three phases: The first phase consists of two content-wise linked patient cases (e.g., in the module “acute abdominal inflammation—the radiological signs of -itis” half of the cohort individually handles a case on sigmoid diverticulitis, while the other half processed a case on cholecystitis). In detail, this case-work phase is conducted by each student using his/her own “workstation” desktop computer equipped with a case-based assessment software for imaging data (VQuest, Utrecht, NL, https://vquest.nl/) providing patient information (anamnesis, laboratory values, etc.) as well as full imaging datasets of ultrasound, X-ray, CT and/or MRI examinations, which can be regularly processed regarding scrolling of image stacks or adjustments to contrast or zooming. For each progressively evolving case, students are required to answer free-text, multiple-choice, and marker questions in VQuest. Questions include competencies in the choice of imaging modality, marking of organs or pathologies, describing and staging of detected pathologies as well as next (therapeutic) steps. An experienced, board-certified radiologist serves as a supervisor and is readily available to students throughout the individual working phase to address significant challenges or questions (approximately one supervisor per 15 students). The second phase of each module contains a supervisor-accompanied small group discussion (about 15 students per group each working on the same case), where all test questions are discussed and true answers presented. This phase is technologically supported by the PRISMA-learning dashboard software [15, 16]. The PRISMA-learning dashboard presents the students’ responses in a structured and anonymous way on a large screen and thus forms the basis of group discussion. During this phase, the supervisor actively engages wrong answers (labels, free text, etc.) anonymously and resolves all remaining open questions. The third phase of each module consists of a short (15 min) concise lecture to the entire cohort of students on the module’s topic and key learning objectives. The primary objective of the lectures is to teach skills in structured description and to identify common radiological patterns in imaging data, rather than to provide a catalog of disease-specific information. The eight modules are tailored in accordance with the national competency-based learning objective catalog and recommendations by the National Radiological Society on teaching contents for undergraduates as well as the Curriculum for Undergraduate Radiological Education by the ESR [7–9] and comprise a spectrum of hepatopancreaticobiliary, endocrinology, and gastrointestinal radiology.

To ensure a consistent learning experience across all modules for each group, all supervisors participate in a training session prior to the course. This session covers learning and educational objectives, the structure of each module and case, and the technical aspects of the course. Supervisors are also provided with a user manual that includes the schedule, group format, software instructions, and educational content, as well as case-specific questions, answers, and lecture materials. Overall, students engage in 6.5 h of educational activities in one day, excluding breaks and a 15-min technical orientation. No other mandatory or optional courses were scheduled for students on this day.

### Study design

In this study, all medical students (*n* = 252) attending the new case- and competency-based abdominal radiology day course between November 2023 to June 2024 (two semester cohorts) at a German medical faculty received a voluntary questionnaire before (12 items) and after (20 items) the course (Supplementary Data 1). The custom-made questionnaire comprised items regarding (1) the perceived importance of abdominal radiological content, (2) the perceived gain of competence in the choice of an imaging modality under given medical information, in locating abdominal organs and judging common abdominal pathologies in radiological imaging data (ultrasound, X-ray, CT and MRI), (3) the perceived fun while learning in the new case- and competency-based format, (4) the interest to pursue a career in radiology, and (5) the pedagogical style of the new course. Questions were either rated by a metric scale from 1 (strongly disagree) to 10 (strongly agree) or dichotomic (yes/no). Metric scale values of 1–2.5 were rated as “strong disagreement,” 2.6–4.5 as “disagreement,” 4.6 to 5.5 as “neutral,” 5.6 to 7.5 as “agreement” and 7.6 to 10.0 as “strong agreement”.

Additionally, all students completed a faculty-mandatory overall course evaluation at the end of the semester 2 months after the case-based abdominal radiology day course, consisting of a continuous visual rating scale from 0 to 100 (0 worst, 100 best) and a free-text option. This overall evaluation was also available from a cohort of students who had previously (May 2022 to June 2023) attended “the old” format of traditional lectures (*n* = 169) and/or seminars (*n* = 234) before the implementation of the new course. The study protocol was exempted from full review by the local ethics board (reference number: 2024-773-f-N).

### Statistical analysis

Data are presented as totals and percentages or mean and standard deviation (SD), as appropriate. The inclusion criterion for all evaluations was a mandatory attendance rate of over 90%. Statistical analysis was conducted using SPSS Statistics version 29 (SPSS Inc., Chicago, IL, USA). A two-sided unpaired Student’s *t*-test was used for mandatory evaluations, while a paired Student’s *t*-test analyzed voluntary questionnaires before and after the course. Cohen’s *d* assessed effect size, with values interpreted as small (0.2), medium (0.5), or large (0.8). Comparisons were performed per item without combining items into a scale. Missing data were handled using pairwise deletion, and a heatmap analysis suggested the data were missing at random. *p*-values < 0.05 were considered statistically significant.

## Results

### Study cohort

In total, voluntary questionnaires from the new case-and competency-based course from *n* = 235 students were received, resulting in a response rate of 93.3%. Students had a mean age of 23.5 ± 2.6 years, while *n* = 64 (27.2%) students were male, *n* = 170 (72.3%) were female, *n* = 1 (0.4%) student did not report gender, and none identified as diverse. Most of the students (*n* = 188, 80.0%) had no prior education or academic degree before entering medical studies, while *n* = 40 (17.0%) had completed prior education (*n* = 24, 10.2% nursing or medical assistant; *n* = 11, 4.7% paramedics, *n* = 7, 3.0% other) and *n* = 7 (3.0%) had received an academic degree. In total, *n* = 22 (9.4%) datasets of voluntary questionnaires before and/or after the course were (partially) incomplete and thus excluded from paired statistical analysis.

### Perceived importance of training in abdominal radiology

**Fig. 2:**
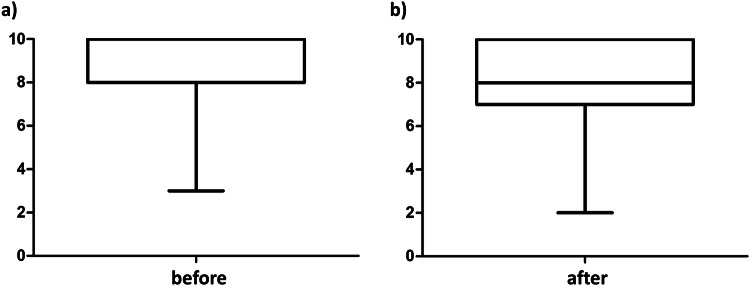
Perceived importance of abdominal radiology teaching. Rating (0—strongly disagree to 10—strongly agree) of medical students. **a** Before the course regarding the importance of learning about abdominal radiology and (**b**) after the course regarding the importance of learned content for the future medical career

First, we evaluated how students judged the importance of receiving teaching in abdominal radiology during their medical studies. Before receiving any teaching, students strongly agreed, with a mean ± SD score of 9.0 ± 1.3, that knowledge of abdominal radiology is important for the profession of a medical doctor independent of later specialization (Fig. 2a). In this context, after the course, students strongly agreed with a score of 8.2 ± 1.7 that the learned content will be beneficial for their future practice (Fig. 2b).

Thus, receiving dedicated radiological training is very important to today’s medical students, and the new case- and competency-based teaching yields a high agreement in perceived importance.

### Perceived gain of competence in abdominal radiology

**Fig. 3:**
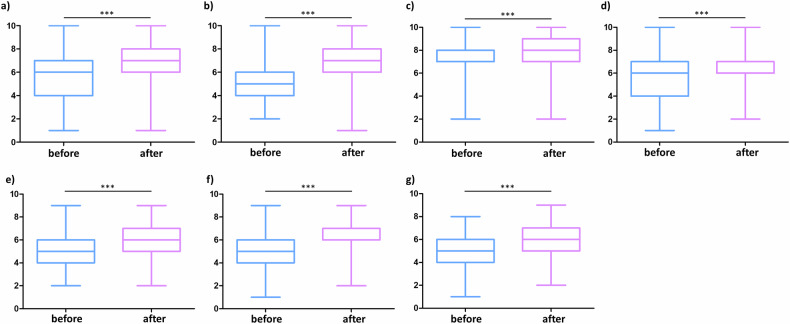
Perceived gain in competence by case- and competency-based learning. Rating (0—strongly disagree to 10—strongly agree) of medical students before and after the course of the perceived competence in (**a**) the knowledge of imaging modalities available for abdominal imaging, (**b**) reasoned decision-making to choose an imaging modality in a defined clinical scenario, (**c**) locating abdominal organs in abdominal imaging datasets; and description and assessment of common abdominal pathologies in (**d**) ultrasound, (**e**) X-ray, (**f**) CT or (**g**) MRI datasets. ***p < 0.001

**Fig. 4:**
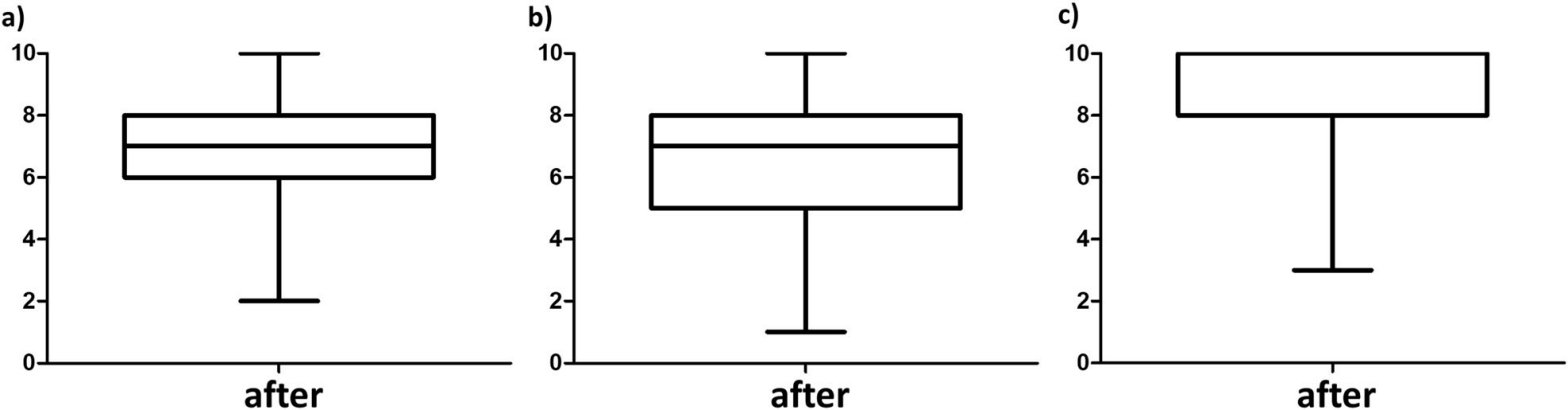
Perceived fun during case- and competency-based learning. Rating (0—strongly disagree to 10—strongly agree) of medical students after the course regarding (**a**) the interest and (**b**) the fun during case-based work. **c** Medical students also rated if the new case- and competency-based format was more fun compared to traditional lectures and seminars

**Fig. 5:**
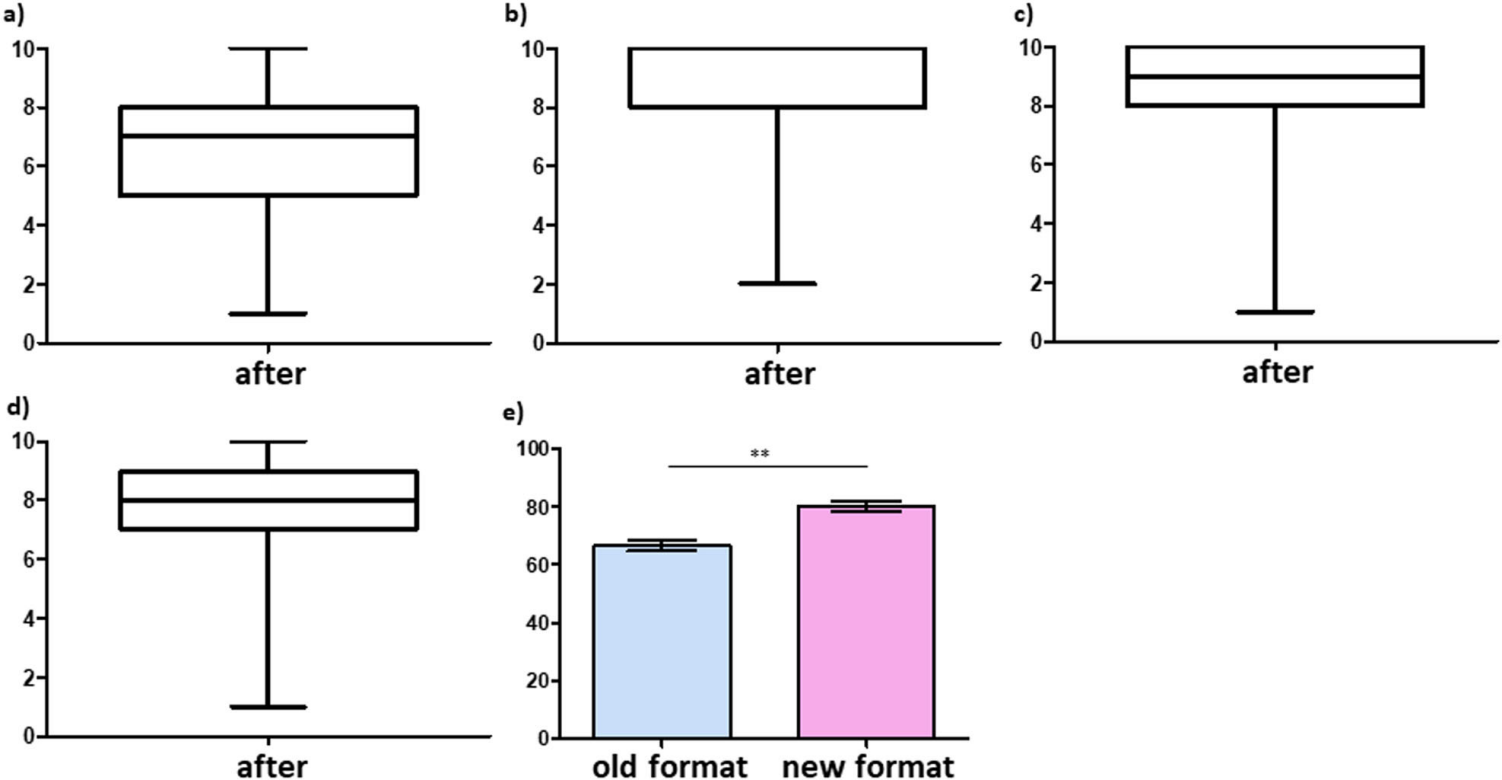
Students’ pedagogical perceptions of the new case- and competency-based teaching approach. Rating (0—strongly disagree to 10—strongly agree) of medical students after the course regarding the additional value compared to traditional lectures and seminars of the (**a**) eLearning module, (**b**) individual workstation-based case-work, (**c**) supervisor mediated small-group case discussion and (**d**) the concise lecture for learning success. **e** Overall rating (0—worst to 100—best) of medical students receiving the new case- and competency-based format compared to the rating of students that had received traditional lectures and seminars prior to the transformation of the teaching format. **p < 0.01

Second, we evaluated the perceived gain in competence of medical students in choosing an imaging modality, as well as image description and analysis, for common abdominal diseases. Detailed numbers of all tested items can be found in Table 1. Briefly, the perceived gain in competence significantly (all *p*-values < 0.001) improved in (1) knowledge of imaging modalities for abdominal imaging (Cohen’s *d* = 0.79, Fig. 3a), (2) reasoned decision-making for choosing imaging modalities (*d* = 0.95, Fig. 3b), (3) locating abdominal organs (*d* = 0.27, Fig. 3c), (4) assessing abdominal pathologies in ultrasound (*d* = 0.62, Fig. 3d), (5) X-ray (*d* = 0.80, Fig. 3e), (6) CT (*d* = 0.86, Fig. 3f), and (7) MRI (*d* = 0.92, Fig. 3g). The strongest effects of the course were seen for reasoned decision-making in choosing an imaging modality in a given clinical scenario (*d* = 0.95), and in image interpretation of more complex, cross-sectional modalities like CT and MRI with effect sizes of *d* = 0.86 and *d* = 0.92, respectively.

**Table 1:** Perceived gain in competence

	Before course	After course	Mean difference	*p*-value	Cohen’s *d* (95% CI)
Knowledge of imaging modalities	5.7 ± 1.7	6.8 ± 1.5	+1.1	< 0.001	0.79 (0.63–0.94)
Reasoned decision-making for choice of modality	5.1 ± 1.7	6.4 ± 1.6	+1.3	< 0.001	0.95 (0.78–1.11)
Locating abdominal organs	7.4 ± 1.5	7.8 ± 1.4	+0.4	< 0.001	0.27 (0.13–0.42)
Description and assessment of pathologies in ultrasound	5.5 ± 1.7	6.5 ± 1.4	+1.0	< 0.001	0.62 (0.47–0.78)
Description and assessment of pathologies in X-ray	5.0 ± 1.6	6.2 ± 1.5	+1.2	< 0.001	0.80 (0.64–0.96)
Description and assessment of pathologies in CT	5.1 ± 1.6	6.4 ± 1.4	+1.3	< 0.001	0.86 (0.70–1.03)
Description and assessment of pathologies in MRI	4.8 ± 1.5	6.1 ± 1.5	+1.3	< 0.001	0.92 (0.75–1.08)

### Perceived fun during the new case- and competency-based course

Third, we analyzed the students’ perceived enjoyment while learning abdominal radiology in a case- and competency-based format as implemented in this study since it has been shown that enjoyment is associated with deep learning improving maintenance of the educated content [17]. Here, students agreed that the description and analysis of findings in radiological imaging data was interesting (6.9 ± 2.0, Fig. 4a) and made fun (6.6 ± 2.1, Fig. 4b). Additionally, students strongly agreed (8.9 ± 1.5, Fig. 4c) that the new case- and competency-based format was more fun than a traditional radiology lecture or seminar.

### The interest to pursue a career in radiology

Fourth, we evaluated if the case- and competency-based course changed the career plans of medical students regarding their future specialization (Supplementary Fig. 1a–c). Here, medical students agreed that an internship in a radiology department during medical studies would be of interest (6.0 ± 2.9), while this agreement was slightly lower after the course (5.6 ± 2.9, *p* = 0.002, Cohen’s *d* = 0.23 95% CI [0.09–0.37]). However, after the course, students had unchanged disagreement with the interest to absolve a part of the last practical year of medical studies in radiology (3.9 ± 2.7) or to pursue a residency in radiology (3.2 ± 3.3) as compared with before the course (4.0 ± 2.6, *p* > 0.05, *d* = 0.16 95% CI [0.01–0.30], or 3.2 ± 2.4, *p* > 0.05, *d* = 0.08 95% CI [0.06–0.22], respectively).

Thus, despite a slight decrease in students’ interest in pursuing an internship in radiology during their studies (*p* = 0.002, Cohen’s *d* = 0.23), which indicates a small effect, the new course had no significant impact on their interest in completing part of their final practical year or pursuing a residency in radiology.

### The pedagogical style of the new case- and competency-based course

Fifth, we analyzed students’ perception of the chosen format for the new case- and competency-based radiology course. Here, students agreed (6.6 ± 2.0, Fig. 5a) that the eLearning module of the first part helped to learn about abdominal imaging in a clinical context. Further, the students strongly agreed (8.8 ± 1.6, Fig. 5b) that the independent case work (scrolling, marking in imaging datasets, etc.) has more supported learning about abdominal radiology than a traditional lecture or seminar. However, besides the first self-study phase, the students strongly agreed (8.5 ± 2.0, Fig. 5c) that the second supervisor-accompanied small group discussion phase was an important complement to ensure learning success and solve open-ended questions. Interestingly, next to the modern design of the first- and second case- and competency-based phase, the students still agreed (7.5 ± 2.1, Fig. 5d) that the concise summarizing lectures were a valuable addition for the overall learning success.

Furthermore, we compared the mandatory overall evaluation of medical students that absolved the new case- and competency-based course with the control group of medical students that had previously received the same teaching content via traditional lectures and seminars. Here, students’ rating of the new case- and competency-based format (80.2 ± 1.7) was significantly better than the rating of traditional lectures and seminars (66.2 ± 1.9, *p* = 0.01, Fig. 5d).

Last, we also analyzed the evaluations’ free-text comments on the new case- and competency-based course. In total, the 235 students formulated 152 free-text comments (free-text response rate of 64.7%), which may have contained positive or negative feedback or both. The most frequent comment for improvement was, that the educational day was too long for a significant part of students to keep up the concentration for effective learning (*n* = 70 comments, 46.1% of comments, 29.7% of students). Further, comments suggested that slide handouts of the short lectures (*n* = 24, 15.8% or 10.2%, respectively) be provided, which had already been implemented after the first course and therefore resulted in no further comments in this regard. Additionally, students requested direct software feedback with the correct/incorrect answers, in addition to the discussion with the supervisor (*n* = 24, 15.8% or 10.2%, respectively). Next to other minor comments not related to the concept of the course (*n* = 9, 5.9% or 3.8%, respectively), some students (*n* = 12, 7.9% or 5.1%, respectively) complained about the loud acoustics in the course room, especially during the group discussion phase. Positive free-text feedback mainly included the overall concept (*n* = 48, 31.6% or 20.0%, respectively), motivation and pedagogical skills of the supervisors (*n* = 19, 12.5% or 8.1%, respectively), and the time schedule (*n* = 7, 4.6% or 3.0%, respectively). Further, an impressive majority (99.5%) of medical students voted to continue the newly implemented case- and competency-based course in the future.

## Discussion

In this study, we demonstrated that receiving training in radiology is important to medical students and that case- and competency-based teaching meets that need, enhances students’ perceived abilities to select imaging modalities suited to specific clinical scenarios, to identify, describe and evaluate common abdominal pathologies, and increases their enjoyment in learning radiology. Current national curricula and society recommendations emphasize the importance of competency-based learning approaches in medical education and especially in radiology due to its continued increase in importance for disease diagnostics, therapy planning and treatment monitoring across all disciplines [7–9, 18–20]. Initial reports have highlighted the positive impact of competency-focused, practical courses in radiology using multimedia-enhanced formats, e.g., applying DICOM viewers [14, 21]. Additionally, also other disciplines have reported positive experiences when applying case-based learning, including anatomy, surgery, neurosciences or medical oncology [13, 22–27]. In our study, we incorporated e-testing tools like VQuest, which enhance student interaction with cases by providing structured case information and enabling diagnostic or therapeutic decision-making questions by embedding various imaging modalities with their strengths and limitations in real-life clinical scenarios [14]. Students can not only view, label, or provide written descriptions of images, but their responses can also be displayed digitally and discussed anonymously, creating a safe environment for resolving misunderstandings and addressing open questions [15]. To further promote this interaction, unlike fully eLearning-based or unsupervised formats [12, 28], our course model ensures the presence of an experienced radiologist on-site during case work, supporting small group discussions and providing a concise lecture summarizing key theoretical content. Interestingly, our data shows that especially the on-site combination of modern case- and competency-based individual work with group discussions and lectures (an “old-fashioned” teaching format) was appreciated more, than online-only approaches such as the eLearning module alone. Although online-only approaches have shown to be effective [22], our results on the students’ perception may favor a comprehensive approach addressing the students’ desire for accessible theoretical knowledge and expert guidance within a case- and competency-based learning environment, avoiding potential cognitive overload and fostering different learning strategies such as independent learning or supportive instruction [6, 21, 29–32].

Nevertheless, the new case- and competency-based format offers opportunities for further improvement. In particular, the course’s current structure, with 6.5 h of training in a single day, should be reconsidered. To optimize engagement, it may be beneficial to divide the course into two half-day sessions or streamline content where feasible. Additionally, noise levels during group discussions in the seminar room could be reduced by implementing acoustic barriers, additional rooms or using audio guide hardware. Additionally, from a specialty perspective, the course format did not foster student interest in pursuing a career in radiology, a considerable point in today’s shortage of personnel. However, this hope may be too much to expect from a single-day course and should be subject to future studies assessing a complete case- and competency-based radiological curriculum across the entire medical degree program. In this context, it is important to note that the presented teaching course does not require expensive equipment, such as full radiological workstations. Instead, it utilizes user-friendly imaging and testing software that can be run on standard desktop computers in typical computer labs, after clinical case examples have been prepared by the radiologist. This makes the course easy to implement at other institutions. Additionally, the course format is highly adaptable and can be applied to other fields of radiology beyond abdominal imaging [15], designing a full case- and competence-based radiological curriculum aligning with the current curricula established by professional societies [7, 9].

Our study is subject to limitations. First, our study relied exclusively on students’ perceptions of the new teaching format, gathered through a self-reported questionnaire. This approach introduces the potential for common method bias which may be enhanced by our design of pre-post testing in the same group of students. However, in curricular medical training, it is not possible to run a randomized 2-group study for ethical reasons. Second, to gain a more objective assessment of the case- and competency-based teaching format and its effectiveness regarding gained knowledge and skills, future studies should analyze outcomes such as test scores on (national) exams or case analysis skills [24, 33]. Third, this study did not aim to assess long-term outcomes, such as the clinical radiological competence of residents in radiology or other specialties who participated in the new case- and competency-based course during their medical studies. Addressing the ultimate goal of medical education—producing better healthcare providers capable of meeting future challenges in the rapidly evolving field of medicine—requires further research to evaluate these long-term impacts. However, it is well-documented that enjoyment during learning—though inherently subjective, as captured in our survey—is critical for fostering interest in the medical field and shaping professional identity [12]. Thus, analysis of the subjective student perception remains essential when evaluating and implementing new instructional models.

In conclusion, this study highlights that, from the students’ perspective, case- and competency-based radiology teaching significantly enhances skill acquisition, learning outcomes, and enjoyment—factors crucial for improving the effectiveness of medical education and shaping both the future of radiology as a specialty and the quality of medical practice for tomorrow’s physicians.

## Supplementary information

ELECTRONIC SUPPLEMENTARY MATERIAL

## Data Availability

All data available are presented within the manuscript; the corresponding author is available for data sharing upon reasonable request.

## Abbreviations

CT: Computed tomography
MRI: Magnetic resonance imaging
SD: Standard deviation
