# Supplementary material for: Students’ perspective on new teaching concepts for medical studies: case- and competency-based learning in radiology
Source: Insights Imaging. 2025 Feb 6;16:31. doi: 10.1186/s13244-025-01909-7 (PMC11803062; doi:10.1186/s13244-025-01909-7)
Supplement: Supplementary file 1 — ELECTRONIC SUPPLEMENTARY MATERIAL [file 13244_2025_1909_MOESM1_ESM.pdf]

Students’ perspective on new teaching concepts for medical studies: Case- and competency-based learning in radiology

ELECTRONIC SUPPLEMENTARY MATERIAL

Supplemental Methods

Curriculum and the traditional teaching concept

Medical students in Germany enter medical school after high-school without any graduation programs. In detail, medical school is divided in a first “pre-clinical” stage consisting of biomedical basics and anatomy for two years completed by a federal board examination, and a second “clinical” stage consisting of a broad spectrum of clinical content for four years (with the last year comprised of a practical year). The “clinical” stage is again completed by a written federal board exam after three years and an oral clinical-practical federal board exam after the fourth year. Each year of the medical school consists of two semesters, while each semester in the “clinical stage” is usually related in terms of a defined content, e.g. with regards to the studied organ systems such as the chest (cardiology, pneumology, cardiothoracic surgery etc.) or abdomen (gastroenterology, visceral surgery etc.). Commonly, cross-field disciplines like radiology provide the related content in a timewise distributed manner across the semester using traditional lectures and seminars. At our medical faculty, hepatopancreaticobiliary and gastrointestinal abdominal clinical contents are teached in the third semester of the clinical stage (which thus equals seventh semester of medical school).

Traditionally, radiology at our medical school was taught primarily through lectures and seminars. In detail, the voluntary lectures consisted of a 45 to 90 min frontal teaching-based talk by an experienced and board-certified radiologist addressing the entire semester cohort of medical students at once. Learning techniques were primarily based on PowerPoint (Microsoft Corporation, Redmond, USA) slides containing radiological image data and / or videos as well as theoretical information prepared as bullet points. Additionally, medical students were able to join the voluntary lectures either on-site or online via Zoom (Zoom Video Communications, San Jose, USA) broadcast with the ability to submit questions and interact with the lecturer. Seminars were structured as 90 min session for groups of about 20 medical students where PowerPoint (Microsoft Corporation, Redmond, USA) slights with mainly an image-based content on abdominal diseases was discussed with the group of students by an experienced and board-certified radiologist. The format enabled for lively discussion about the projected images, but “active participation” is not fostered by respective pedagogical strategies and thus was usually carried out only by a portion of attending medical students. Seminars were mandatory (at least 90% attendance rate) for each student, while attendance was again possible either on-site or online. In total, the third semester of the “clinical” stage on hepatopancreaticobiliary and gastrointestinal abdominal diseases consisted of 5 lectures à 45 minutes and 1 seminar à 90 minutes in radiology offered to each student, while the time interval

between lectures and the seminar may have strongly differed due to varying timetables for each student.

Supplementary data 1 - Questionnaire

Survey – Case- and Competency-based Course in Abdominal Radiology

Part 1 (before on-site course)

Age: _________

sex: **m** ☐ **f** ☐ **d** ☐ **seat number:** ___________

Question 1: I have completed vocational training or an academic degree either prior to or alongside my medical studies.

o No

o Nursing, Medical Assistant (MFA), Medical Laboratory Assistant (MTLA)

o Radiology Technician / Medical Technical Radiology Assistant (MTRA / MTR)

o Other Vocational Training

o Bachelor's Degree (Health Professions)

o Master's Degree (Health Professions)

o Other Training: ______________________________

o Other Degree: ______________________________

Question 2: I believe that knowledge about abdominal radiology is important for my future career as a physician.

Please indicate the answer on the following scale (1 = strongly disagree, 10 = strongly agreee):

disagree	0	0	0	0	0	0	0	0	0	0	agree
	1	2	3	4	5	6	7	8	9	10	

Question 3: I am familiar with the various imaging modalities for radiological examinations of the abdomen, including their advantages, disadvantages, and risks.

Please indicate the answer on the following scale (1 = strongly disagree, 10 = strongly agree):

disagree	0	0	0	0	0	0	0	0	0	0	agree
	1	2	3	4	5	6	7	8	9	10	

Question 4: I feel capable of selecting between different imaging modalities for abdominal radiological examinations in a clinical context.

Please indicate the answer on the following scale (1 = strongly disagree, 10 = strongly agree):

disagree	0	0	0	0	0	0	0	0	0	0	agree
	1	2	3	4	5	6	7	8	9	10	

Question 5: I feel capable of identifying various abdominal organs in radiological imaging datasets.

Please indicate the answer on the following scale (1 = strongly disagree, 10 = strongly agree):

disagree	0	0	0	0	0	0	0	0	0	0	agree
	1	2	3	4	5	6	7	8	9	10	

Question 6: I feel capable of describing and assessing common abdominal pathologies in sonographic images.

Please indicate the answer on the following scale (1 = strongly disagree, 10 = strongly agree):

disagree	0	0	0	0	0	0	0	0	0	0	agree
	1	2	3	4	5	6	7	8	9	10	

Question 7: I feel capable of describing and assessing common abdominal pathologies in X-ray images.

Please indicate the answer on the following scale (1 = strongly disagree, 10 = strongly agree):

disagree	0	0	0	0	0	0	0	0	0	0	agree
	1	2	3	4	5	6	7	8	9	10	

Question 8: I feel capable of describing and assessing common abdominal pathologies in CT scans.

Please indicate the answer on the following scale (1 = strongly disagree, 10 = strongly agree):

disagree	0	0	0	0	0	0	0	0	0	0	agree
	1	2	3	4	5	6	7	8	9	10	

Question 9: I feel capable of describing and assessing common abdominal pathologies in MRI scans.

Please indicate the answer on the following scale (1 = strongly disagree, 10 = strongly agree):

disagree	0	0	0	0	0	0	0	0	0	0	agree
	1	2	3	4	5	6	7	8	9	10	

Question 10: I can envision completing an internship in radiology during my studies.

Please indicate the answer on the following scale (1 = strongly disagree, 10 = strongly agree):

disagree	0	0	0	0	0	0	0	0	0	0	agree
	1	2	3	4	5	6	7	8	9	10	

Question 11: I can envision doing a rotation within the practical year (PY) of my studies in radiology.

Please indicate the answer on the following scale (1 = strongly disagree, 10 = strongly agree):

disagree	0	0	0	0	0	0	0	0	0	0	agree
	1	2	3	4	5	6	7	8	9	10	

Question 12: I can envision pursuing a specialization / residency in radiology after completing my studies.

Please indicate the answer on the following scale (1 = strongly disagree, 10 = strongly agree):

disagree	0	0	0	0	0	0	0	0	0	0	agree
	1	2	3	4	5	6	7	8	9	10	

Survey – Case- and Competency-based Course in Abdominal Radiology

Part 2 (after on-site course)

Age: __________

sex: **m** ☐ **f** ☐ **d** ☐ **seat number:** ____________

Question 1: I believe that the case- and competency-based abdominal radiology course will be beneficial for my future career as a physician.

Please indicate the answer on the following scale (1 = strongly disagree, 10 = strongly agree):

disagree	0	0	0	0	0	0	0	0	0	0	agree
	1	2	3	4	5	6	7	8	9	10	

Question 2: I am familiar with the various imaging modalities for radiological examinations of the abdomen, including their advantages, disadvantages, and risks.

Please indicate the answer on the following scale (1 = strongly disagree, 10 = strongly agree):

disagree	0	0	0	0	0	0	0	0	0	0	agree
	1	2	3	4	5	6	7	8	9	10	

Question 3: I feel capable of selecting between different imaging modalities for abdominal radiological examinations in a clinical context.

Please indicate the answer on the following scale (1 = strongly disagree, 10 = strongly agree):

disagree	0	0	0	0	0	0	0	0	0	0	agree
	1	2	3	4	5	6	7	8	9	10	

Question 4: I feel capable of identifying various abdominal organs in radiological imaging datasets.

Please indicate the answer on the following scale (1 = strongly disagree, 10 = strongly agree):

disagree	0	0	0	0	0	0	0	0	0	0	agree
	1	2	3	4	5	6	7	8	9	10	

Question 5: I feel capable of describing and assessing common abdominal pathologies in sonographic images.

Please indicate the answer on the following scale (1 = strongly disagree, 10 = strongly agree):

disagree	0	0	0	0	0	0	0	0	0	0	agree
	1	2	3	4	5	6	7	8	9	10	

Question 6: I feel capable of describing and assessing common abdominal pathologies in X-ray images.

Please indicate the answer on the following scale (1 = strongly disagree, 10 = strongly agree):

disagree	0	0	0	0	0	0	0	0	0	0	agree
	1	2	3	4	5	6	7	8	9	10	

Question 7: I feel capable of describing and assessing common abdominal pathologies in CT scans.

Please indicate the answer on the following scale (1 = strongly disagree, 10 = strongly agree):

disagree	0	0	0	0	0	0	0	0	0	0	agree

	1	2	3	4	5	6	7	8	9	10	

Question 8: I feel capable of describing and assessing common abdominal pathologies in MRI scans.

Please indicate the answer on the following scale (1 = strongly disagree, 10 = strongly agree):

disagree	0	0	0	0	0	0	0	0	0	0	agree
	1	2	3	4	5	6	7	8	9	10	

Question 9: I find the radiological description and assessment of imaging studies of the abdomen to be interesting.

Please indicate the answer on the following scale (1 = strongly disagree, 10 = strongly agree):

disagree	0	0	0	0	0	0	0	0	0	0	agree
	1	2	3	4	5	6	7	8	9	10	

Question 10: The radiological description and assessment of imaging studies of the abdomen makes fun.

Please indicate the answer on the following scale (1 = strongly disagree, 10 = strongly agree):

disagree	0	0	0	0	0	0	0	0	0	0	agree
	1	2	3	4	5	6	7	8	9	10	

Question 11: The e-learning module helped me learn about the knowledge of abdominal imaging in a clinical context.

Please indicate the answer on the following scale (1 = strongly disagree, 10 = strongly agree):

disagree	0	0	0	0	0	0	0	0	0	0	agree
	1	2	3	4	5	6	7	8	9	10	

Question 12: Independently working on case studies (scrolling/marking in image datasets) has helped me learn about abdominal radiological examinations in a clinical context more than a traditional lecture or seminar.

Please indicate the answer on the following scale (1 = strongly disagree, 10 = strongly agree):

disagree	0	0	0	0	0	0	0	0	0	0	agree
	1	2	3	4	5	6	7	8	9	10	

Question 13: The small group case discussion with support from the instructors was an important complement to learning with case studies and solving open questions.

Please indicate the answer on the following scale (1 = strongly disagree, 10 = strongly agree):

disagree	0	0	0	0	0	0	0	0	0	0	agree
	1	2	3	4	5	6	7	8	9	10	

Question 14: The concise lectures after each case were a valuable addition to learning about abdominal radiological examinations in a clinical context.

Please indicate the answer on the following scale (1 = strongly disagree, 10 = strongly agree):

disagree	0	0	0	0	0	0	0	0	0	0	agree
	1	2	3	4	5	6	7	8	9	10	

Question 15: I can envision completing an internship in radiology during my studies.

Please indicate the answer on the following scale (1 = strongly disagree, 10 = strongly agree):

disagree	0	0	0	0	0	0	0	0	0	0	agree
	1	2	3	4	5	6	7	8	9	10	

Question 16: I can envision doing a rotation within the practical year (PY) of my studies in radiology.

Please indicate the answer on the following scale (1 = strongly disagree, 10 = strongly agree):

disagree	0	0	0	0	0	0	0	0	0	0	agree
	1	2	3	4	5	6	7	8	9	10	

Question 17: I can envision pursuing a specialization / residency in radiology after completing my studies.

Please indicate the answer on the following scale (1 = strongly disagree, 10 = strongly agree):

disagree	0	0	0	0	0	0	0	0	0	0	agree
	1	2	3	4	5	6	7	8	9	10	

Question 18: The case- and competency-based course on abdominal radiology was more enjoyable for me than a traditional radiology lecture or seminar

Please indicate the answer on the following scale (1 = strongly disagree, 10 = strongly agree):

disagree	0	0	0	0	0	0	0	0	0	0	agree
	1	2	3	4	5	6	7	8	9	10	

Question 19: The case- and competency-based course on abdominal radiology should be retained in the future.

- Yes
- No

Question 20: Please note any criticisms, suggestions for improvement, comments, or praise, etc.

Supplementary Figures

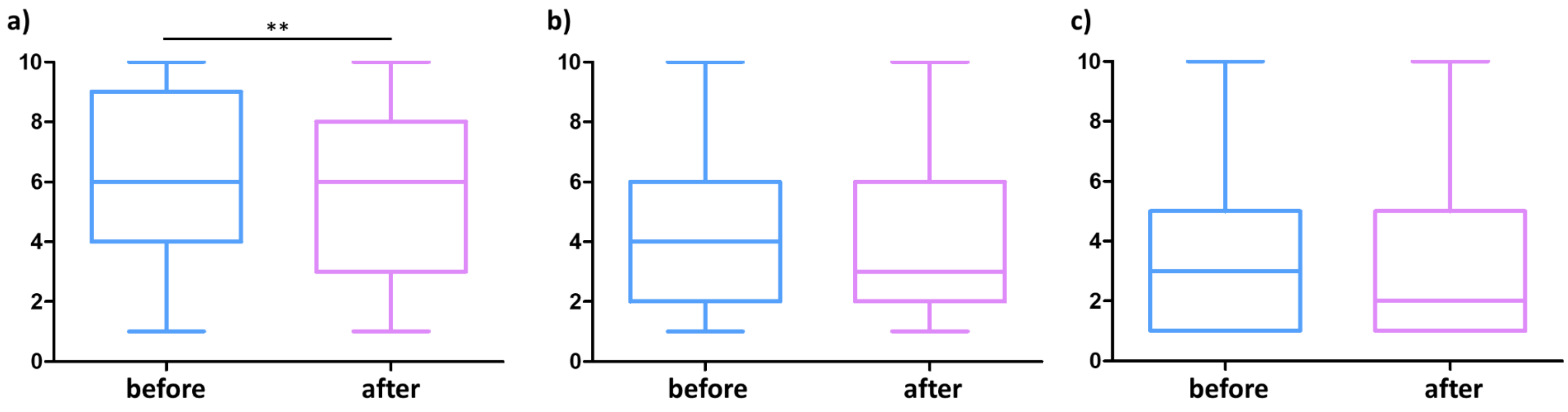

Supplementary Figure 1: The Interest to Pursue a Career in Radiology

Rating (0 – strongly disagree to 10 – strongly agree) of medical students before and after the course regarding the interest **a)** to complete an internship in radiology during medical studies, **b)** to complete a rotation within the practical year of medical studies in radiology, or **c)** to pursue a specialization / residency in radiology after completing medical studies.
